# MiR-23a promotes TGF-β1-induced EMT and tumor metastasis in breast cancer cells by directly targeting CDH1 and activating Wnt/β-catenin signaling

**DOI:** 10.18632/oncotarget.18422

**Published:** 2017-06-09

**Authors:** Fei Ma, Wenjie Li, Chunxiao Liu, Wei Li, Haining Yu, Bo Lei, Yanlv Ren, Zhigao Li, Da Pang, Cheng Qian

**Affiliations:** ^1^ Department of Breast Surgery, Harbin Medical University Cancer Hospital, Harbin, China; ^2^ Translational Medicine Research and Cooperation Center of Northern China, Heilongjiang Academy of Medical Sciences, Harbin, China

**Keywords:** miR-23a, TGF-β1, R-SBE, CDH1, Wnt/β-catenin

## Abstract

TGF-β1-induced epithelial-mesenchymal transition (EMT) has been proved to be associated with metastasis of breast cancer cells. We attempted to detect a novel mechanism that microRNAs mediated the TGF-β1-induced EMT in the process of breast cancer metastasis. Here we reported that the expression of miR-23a was higher in breast cancer cells with high metastasis ability and patients with lymph node metastasis and the treatment of TGF-β1 significantly upregulated the expression of miR-23a in breast cancer cells. We found that miR-23a was upregulated by TGF-β1 post-transcriptionally and Smads directly bound the RNA Smad binding element (R-SBE) of miR-23a. Functional studies showed that inhibition of miR-23a suppressed the TGF-β1-induced EMT, migration, invasion and metastasis of breast cancer both *in vitro* and *in vivo*. In addition, we determined that miR-23a directly targeted and suppressed CDH1, one important gene in EMT phenomenon. Notably, Wnt/β-catenin signaling was activated by the suppression of CDH1 in the miR-23a mediated process of TGF-β1-induced EMT and tumor invasion. These results demonstrate that miR-23a promotes TGF-β1-induced tumor metastasis in breast cancer by targeting CDH1 and activating Wnt/β-catenin signaling. Taken together, our results indicate a novel regulatory mechanism of TGF-β1-induced EMT and suggest that miR-23a might be a potential target in breast cancer therapy.

## INTRODUCTION

At present, tumor metastasis is the leading cause of breast cancer death among women because of its surgically inoperable nature and the resistance to existing therapeutic drugs [1, 2]. Tumor metastasis is modulated by numerous factors, such as angiogenesis, signal transduction and metastasis-related factors [3]. Although much achievement has been made in this field, elucidation of the precise molecular mechanism that governs metastasis remains enigmatic.

Recent studies revealed that epithelial-mesenchymal transition (EMT) played a pivotal role in the process of breast cancer metastasis. EMT is characterized by the loss of epithelial differentiation markers including E-cadherin and the induction of mesenchymal markers such as vimentin and fibronectin [4]. During the EMT process, tumor cells lose cell polarity [5] and the connection between cells becomes loose [6]. This process of EMT is the crucial step for cancer cells to metastasis. TGF-β, a cytokine with multiple biological functions, was first described as an inducer of EMT in normal mammary epithelial cells, and several subsequent studies reported important roles of TGF-β-induced EMT in tumor metastasis [7]. It was reported that TGF-β not only activated the Smad pathway but also alternative effectors, such as mitogen-activated protein kinase, Rho-like GTPases and phosphatidylinositol-3 kinase to induce EMT [8]. Although several studies have focused on the mechanisms of the disintegration of adherens junctions mediated by TGF-β, the detailed elaboration of the mechanisms underlying TGF-β inducing EMT and tumor metastasis remain unclear [9].

Recently, accumulating studies have established a central regulatory role for miRNAs in the development of breast cancer [10]. These small non-coding RNAs can modulate the expression levels of one third of all human protein-coding genes [11]. They can bind to the 3’-untranslated region (UTR) of target mRNAs and cause mRNAs cleave or translational inhibition [12]. It has also been reported that many miRNAs were involved in the process of EMT in cancer cells. MiR-10b, miR-21 and miR-181a have been found to play important roles in the process of TGF-β-induced EMT [13–14]. However, the precise mechanism that microRNAs regulate the TGF-β-induced EMT remains unknown. In the present study, we hypothesized that miR-23a, which was reported associated with breast cancer invasion, might play a regulatory role in the TGF-β-induced EMT in breast cancer.

Wnt/β-catenin signaling is well studied in the process of breast cancer development. Beta-catenin protein can bind to the cytoplasmic domain of E-cadherin (CDH1) and link the adherens junctions to the actin cytoskeleton [15]. Upon activation, β-catenin protein will accumulate in the cytoplasm and disassociate with E-cadherin. Activated β-catenin can translocate into the nucleus and promote the expression of a series target genes which mostly associated with tumor progression [16, 17]. It is reported that Wnt/β-catenin pathway is regulated by multiple factors in breast cancer cells, including microRNAs [18]. Thus, identifying the upstream regulators of Wnt/β-catenin signaling is of important meaning to breast cancer treatment.

In this study, we measured the expression level of miR-23a in breast cancer cell lines and tissues and found that TGF-β1 upregulated the expression of miR-23a. We further demonstrated that the TGF-β/Smad signal-regulated miR-23a post-transcriptionally and confirmed the binding sequences. We showed that inhibition of miR-23a suppressed the TGF-β1-induced EMT, migration, invasion and metastasis of breast cancer via a series of experiments *in vitro* and *in vivo*. Meanwhile, we found that miR-23a acted its function by directly targeting CDH1 to further activate Wnt/β-catenin signaling. Altogether, these data not only associate miR-23a with metastasis of breast cancer but also provide a new therapeutic target for treating breast cancer.

## RESULTS

### MiR-23a expression was higher in patients with lymph node metastasis and metastatic cell lines and the treatment of TGF-β1 upregulated the expression of miR-23a in MCF-7 and MDA-MB-231 cell lines

**Figure 1:**
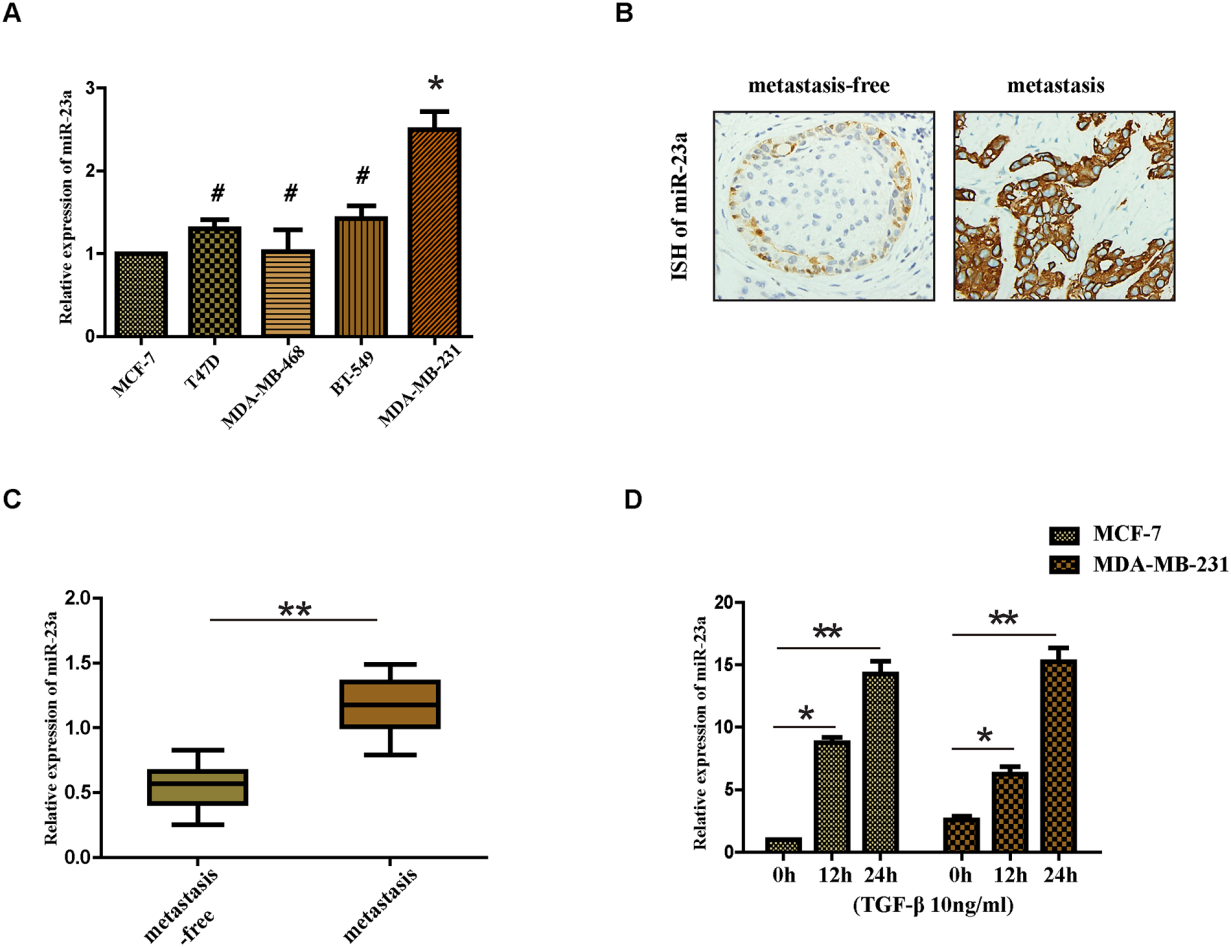
Expression of miR-23a in cell lines and human tissues and the treatment of TGF-β1 upregulates the expression of miR-23a in MCF-7 and MDA-MB-231 cell lines **(A)** Real-time PCR analysis of the expression of miR-23a in different breast cancer cell lines, normalized to GAPDH. **(B)** Analysis of miR-23a *in situ* hybridization(ISH) signal in breast cancer tissues. Representative images are shown (400 magnification). **(C)** Real-time PCR analysis of miR-23a expression in a group of 30 BC patients without lymph node metastasis and matched patients with lymph node metastasis. **(D)** Real-time PCR analysis of the expression of miR-23a in MCF-7 and MDA-MB-231 cells treated with 10ng/ml TGF-β1 for 12h or 24h. *P<0.05, **P<0.01.

We first set out to analysis the expression of miR-23a in breast cancer cells with different metastasis ability, including MCF-7, T47D, MDA-MB-468, BT-549 and MDA-MB-231. We observed that the expression of miR-23a was significantly higher in MDA-MB-231 cells than that in MCF-7 cells (Figure 1A). Consistent with previous study [19], we found that the expression of miR-23a in breast cancer patients with lymph node metastasis was markedly higher compared with that of patients without lymph node metastasis (Figure 1B and 1C), suggesting that miR-23a might function as a tumor promoter. A previous study showed that activation of TGF-β receptor type I led to phosphorylation of receptor-specific SMAD (R-SMAD) proteins and R-SMADs could play a role in the post-transcriptional regulation of microRNAs [16]. Therefore, we next detected the expression of miR-23a in MCF-7 and MDA-MB-231 cells treated with TGF-β1 by qRT-PCR. We found that the miR-23a expression increased significantly in the two cell lines after the treatment with TGF-β1 for 12h and 24h (Figure 1D). The results demonstrate that the treatment of TGF-β1 significantly upregulates the expression of miR-23a in MCF-7 and MDA-MB-231 cell lines.

### TGF-β1 regulated miR-23a post-transcriptionally and the R-SBE sequence was essential for the association of SMAD MH1 domain and miR-23a

**Figure 2:**
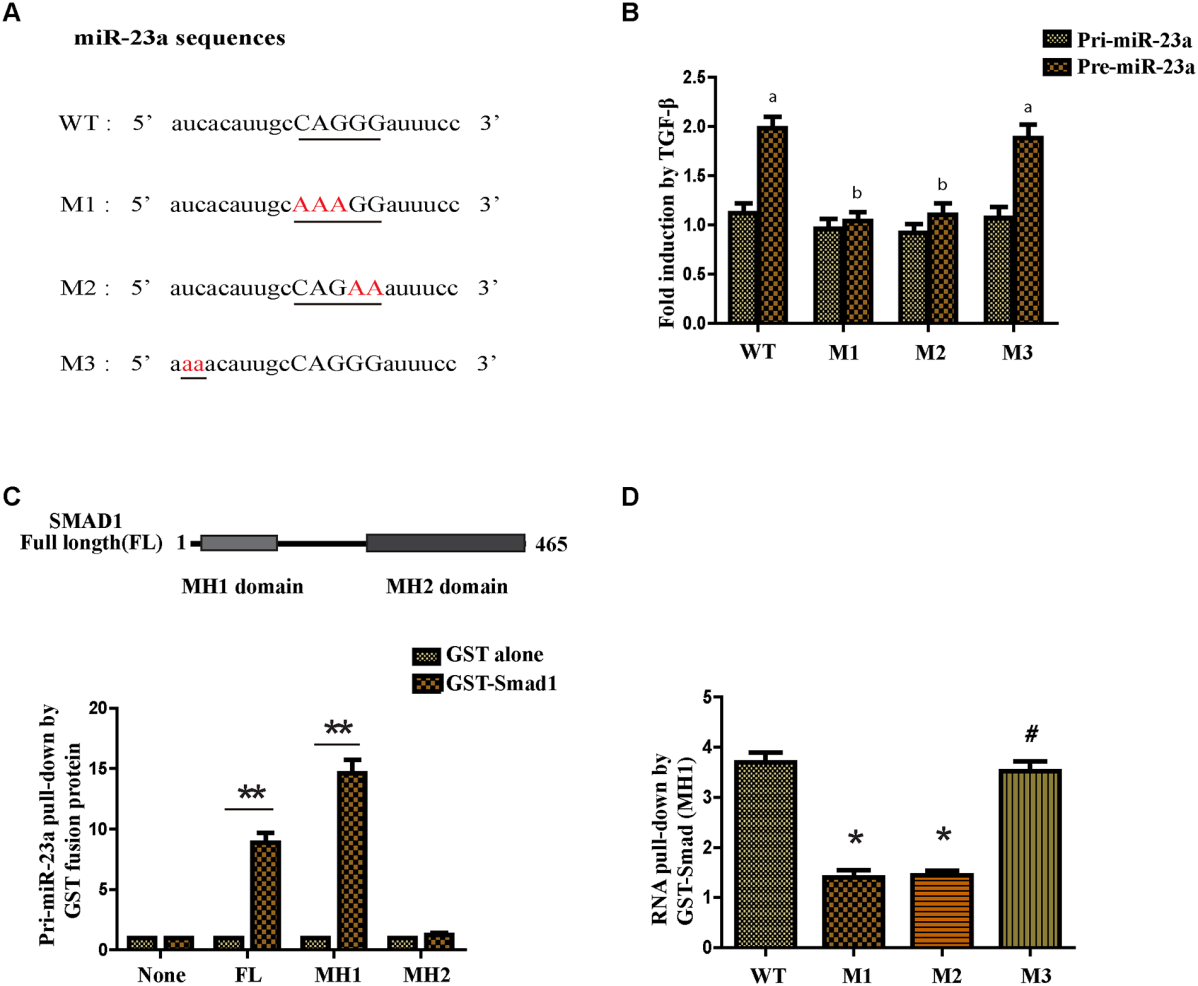
TGF-β1 regulates miR-23a post-transcriptionally and the R-SBE sequence is essential for the association of SMAD MH1 domain and miR-23a **(A)** Schematic diagram of pre-miR-23a wild-type and mutant sequences. Underlined characters indicate the R-SBE sequence found in pre-miR-23a and red highlighted characters indicate mutation introduced. **(B)** MCF-7 cells were transfected with human pri-miR-23a expression constructs, followed by treatment with or without 10ng/ml TGF-β1 for 2h and subjected to real-time PCR analysis using primers to detect exogenous pri-miR-23a or pre-miR-23a, normalized to GAPDH. Fold induction by the TGF-β1 relative to mock treated cells was displayed. Values labeled with different letters differed from one another (P<0.05). **(C)** The schematic diagram of domains of Smad1 protein (upper panel). *In vitro* transcribed wild type pri-miR-23a was mixed with indicated recombinant, sepharose bead-immobilized GST-fusion proteins. Associated RNA was eluted, and subjected to real-time PCR analysis to detect pri-miR-23a. The relative amount of pri-miR-23a pulled down with GST-Smad fusion proteins, normalized to the amount pulled down with GST alone is presented (lower panel). **(D)** *In vitro* transcribed pri-miR-23a constructs were mixed with recombinant GST-Smad1 (MH1) or GST alone and the relative amount of pri-miR-23a transcripts pulled down with GST-Smad1 (MH1) fusion protein was normalized to the amount pulled down with GST alone. *P<0.05, **P<0.01.

It was reported that some microRNAs contain a sequence 5’-CAGAC-3’ or 5’-CAGGG-3’ in the middle of the mature miRNA region and the sequence was similar to the Smad-binding element (SBE) found in the promoter region of TGF-β target genes [16]. Thus it was called RNA Smad binding element (R-SBE). Interestingly, there was a consensus sequence in the stem region of miR-23a. To test if an R-SBE sequence is essential for the interaction between miR-23a and SMAD, we generated mutants to the R-SBE region and a sequence upstream of the R-SBE in the stem region (Figure 2A). Next we generated the PCR primers that were specifically designed for human pri- and pre-miR-23a (WT or mutants) to detect the exogenous pri- and pre-miR-23a transcripts. As expected, the processing of WT pre-miR-23a was enhanced with the treatment of TGF-β1. However, pre-miR-23a induction was abolished when the R-SBE sequence was changed (Figure 2B). Furthermore, unlike the R-SBE mutants, mutants in the sequence upstream of the R-SBE in the stem region did not affect the TGF-β1-induced processing of pre-miR-23a (Figure 2B). Together, these data indicate that the R-SBE is essential for TGF-β-dependent induction of pri-miR-23a processing.

To examine the possibility if the Smads may directly associate with pri-miR-23a, we partially purified recombinant GST-Smad fusion proteins and conjugated them to glutathione S-sepharose beads. We used the recombinant proteins to pull down transcribed pri-miR-23a *in vitro*. The pri-miR-23a co-precipitating with GST-Smad fusion proteins were examined by qRT-PCR analysis (Figure 2C). We found that full-length Smad1 and the MH1 domain of Smad1 were able to pull-down more pri-miR-23a compared with GST protein alone (Figure 2C). However, the carboxyl terminus MH2 domain of Smad1 did not interact with pri-miR-23a (Figure 2C). Again, to determine the role of R-SBE, the MH1 domain of R-Smads was used to pull down pri-miR-23a, WT or mutants. The R-SBE mutants (M1 and M2) exhibited reduced binding to MH1 domain compared to WT pri-miR-23a (Figure 2D). Conversely, mutants in the sequence upstream of the R-SBE exhibited similar binding ability as WT pri-miR-23a (Figure 2D), indicating that the R-SBE is specifically required for R-Smad binding. To conclude, TGF-β1 regulates miR-23a post-transcriptionally, and the R-SBE sequence of miR-23a is essential for the association with Smad MH1 domain.

### Inhibition of miR-23a suppressed the TGF-β1-induced EMT, the migration and invasion ability of breast cancer cells treated with TGF-β1 both *in vitro* and *in vivo*

**Figure 3:**
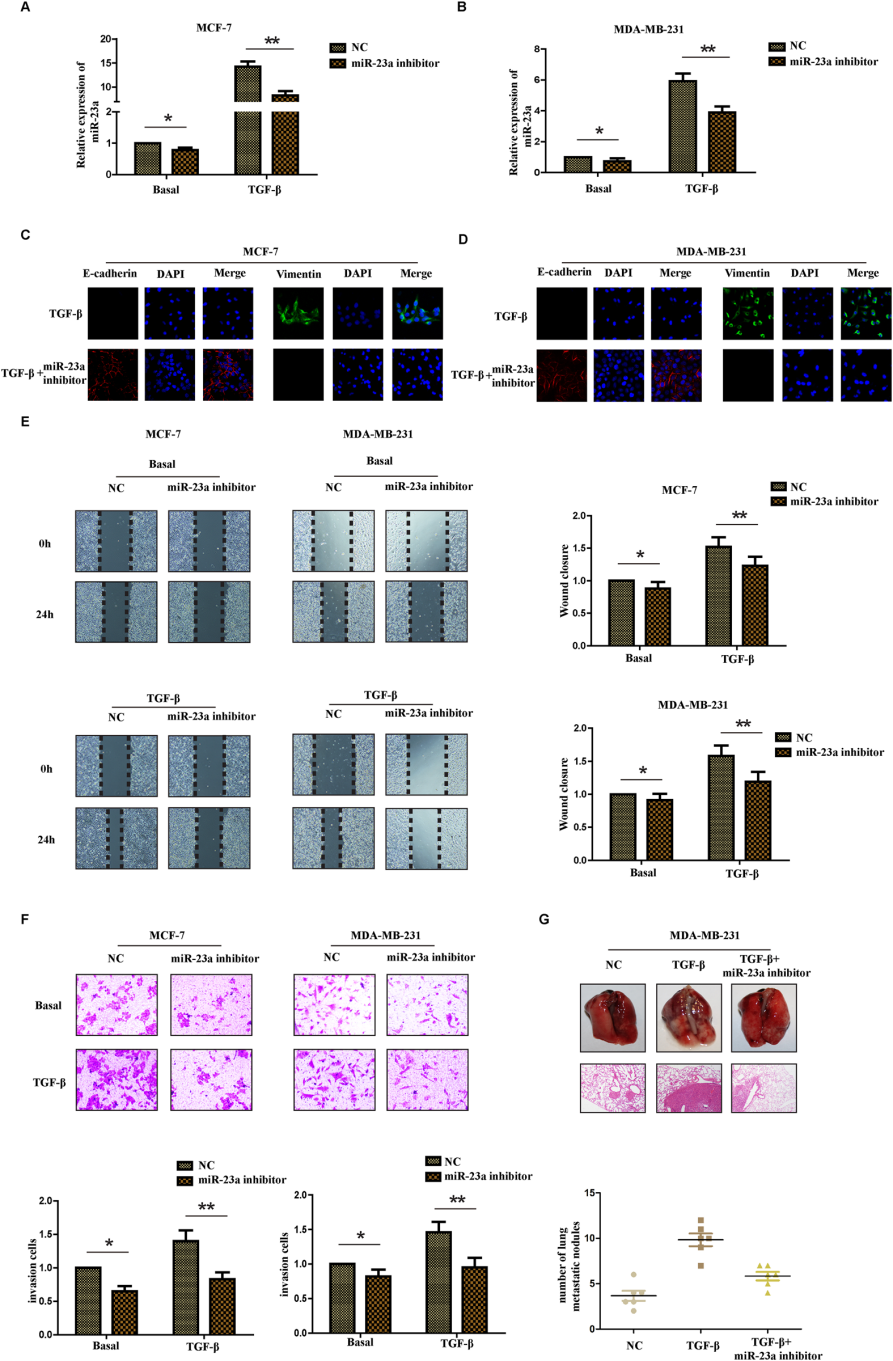
Inhibition of miR-23a suppresses the TGF-β1-induced EMT, the migration and invasion ability of breast cancer cells treated with TGF-β1 Real-time PCR was used to confirm the transfection efficiency of miR-23a in MCF-7 **(A)** and MDA-MB-231 **(B)** cells treated with or without TGF-β1. **(C** and **D)** Representative IF images indicated that miR-23a had an effect on the expression of EMT genes in indicated cells treated with TGF-β1. **(E)** Representative micrograph images of wound healing assay of the indicated cells. Wound closures were photographed at 0h and 24h after wounding. **(F)** Invasion assays of the indicated cells transfected with NC or miR-23a inhibitor. **(G)** Tumor metastasis analysis. Representative images of lung tissues and micrographs of HE staining of metastatic tumor tissues (upper panel). The number of metastatic lung nodules in each group of nude mice (n= 6 per group, lower panel). *P<0.05, **P<0.01.

It is well known that TGF-β1 was first described as an inducer of EMT in normal mammary epithelial cells. To understand the biological effect of miR-23a in the process of TGF-β1-induced EMT, we transfected MCF-7 cells and MDA-MB-231 cells with miR-23a inhibitor. The transfection efficiency was confirmed by detecting the expression of miR-23a in different cell lines with or without TGF-β1 treatment and the inhibitory effect in cells treated with TGF-β1 was more obvious than that in cells without TGF-β1 treatment (Figure 3A and 3B). In addition, there was a marked change in the expression of hallmark EMT genes in miR-23a inhibition cells based on immunofluorescence staining. Transfection of miR-23a inhibitor significantly decreased the expression of vimentin, while increased the expression of E-cadherin in both cell lines (Figure 3C and 3D). To further explore the effects of miR-23a inhibition in the process of TGF-β1-induced EMT in breast cancer cells, we examined the changes of migration and invasion ability of cells treated with or without TGF-β1. Wound healing assay showed that treatment with TGF-β1 for 24hrs markedly increased the migration ability of MCF-7 and MDA-MB-231 cells. Meanwhile, transfection of miR-23a inhibitors significantly decreased the migration ability of the breast cancer cells (Figure 3E). Similarly, Matrigel-coated transwell assays demonstrated that treatment with TGF-β1 for 24hrs obviously increased the invasion ability of MCF-7 and MDA-MB-231 cells. The breast cancer cells transfected with miR-23a inhibitor were also found to have significantly lower rate of invasion than control cells (Figure 3F). The results revealed that down-regulation of miR-23a inhibited the migration and invasion capacity of breast cancer cells treated by TGF-β1 *in vitro*.

To further investigate the metastatic ability of miR-23a-inhibited cells treated with TGF-β1 *in vivo*, control MDA-MB-231 cells, MDA-MB-231 cells with TGF-β1 treatment for 24h and miR-23a-inhibited cells with TGF-β1 treatment were injected into the lateral tail vein of nude mice. As shown in Figure 3G, mice injected with TGF-β1-treated cells exhibited more visible metastatic nodules in the lung than the control cells. Meanwhile, the miR-23a-inhibited cells with TGF-β1 treatment exhibited less metastatic capacity than the cells simply treated with TGF-β1. The results were confirmed by hematoxylin-eosin staining and statistical analysis, indicating that down-regulation of miR-23a inhibited the metastatic capacity of breast cancer cells treated with TGF-β1 *in vivo*. Taken together, our findings suggest that down-regulation of miR-23a inhibits breast cancer cells treated with TGF-β1 invasion and metastatic potentialities.

### CDH1 was a direct target of miR-23a

**Figure 4:**
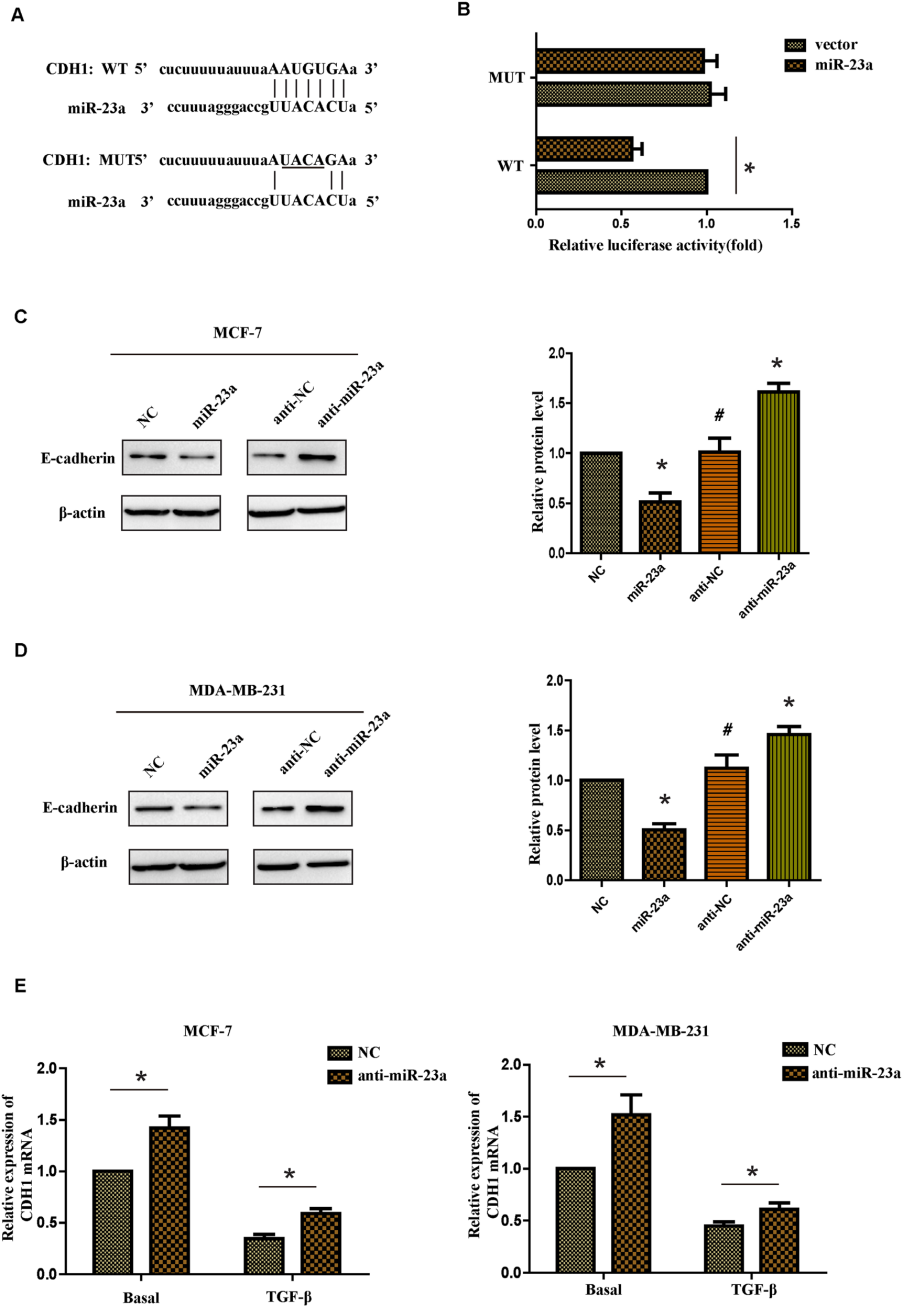
CDH1 is a direct target of miR-23a **(A)** Luciferase reporter plasmids containing the putative wild-type or mutant miR-23a binding sequence in 3’-UTR of CDH1 mRNA. **(B)** HEK293T cells were co-transfected with a control vector or miR-23a and a luciferase reporter construct containing the wild-type or mutant CDH1 3’-UTR. The results were normalized, and the luciferase activity of the control was set to 1. **(C** and **D)** Effects of miR-23a dysregulation of CDH1 expression were detected by western blot analysis in MCF-7 and MDA-MB-231 cells. **(E)** Real-time PCR analysis of CDH1 mRNA expression of the NC and anti-miR-23a-transfected cells treated with or without TGF-β1. *P<0.05, **P<0.01.

To further investigate the mechanism of miR-23a regulating the migration and invasion of breast cancer cells, it is necessary to determine an mRNA target of miR-23a. The target prediction programs miRWalk and TargetScan were used to predict the potential target of miR-23a. CDH1 attracted our attention because it played an important role in the TGF-β-induced EMT and it contained a possible binding site of miR-23a in the 3’-UTR (Figure 4A). To confirm if CDH1 was the direct target of miR-23a, we constructed a luciferase reporter plasmid containing CDH1-3’UTR with a conserved miR-23a binding site and CDH1-3’UTR with a mutated miR-23a binding sequence. The binding ability of miR-23a to CDH1-3’UTR was detected by dual-luciferase reporter assay. It was found that co-expression of miR-23a significantly inhibited the firefly luciferase reporter activity of the wild-type CDH1-3’UTR, but not the activity of the mutant CDH1-3’UTR constructs (Figure 4B). The results showed that miR-23a inhibited the expression of CDH1 by directly binding to the 3’-UTR. Western blot assays indicated that the protein level of CDH1 was markedly reduced in miR-23a transfected cells, whereas anti-miR-23a transfection obviously increased expression of the CDH1 protein in MCF-7 and MDA-MB-231 cells (Figure 4C and 4D). Similar results were found in the mRNA level in both cell lines. Meanwhile, indicated cells treated with TGF-β1 markedly reduced the basal mRNA level of CDH1 (Figure 4E). In conclusion, our results indicate that miR-23a directly decreases CDH1 expression in breast cancer cells.

### CDH1 critically contributed to the pro-metastatic function of miR-23a in breast cancer cells with TGF-β1 treatment

**Figure 5:**
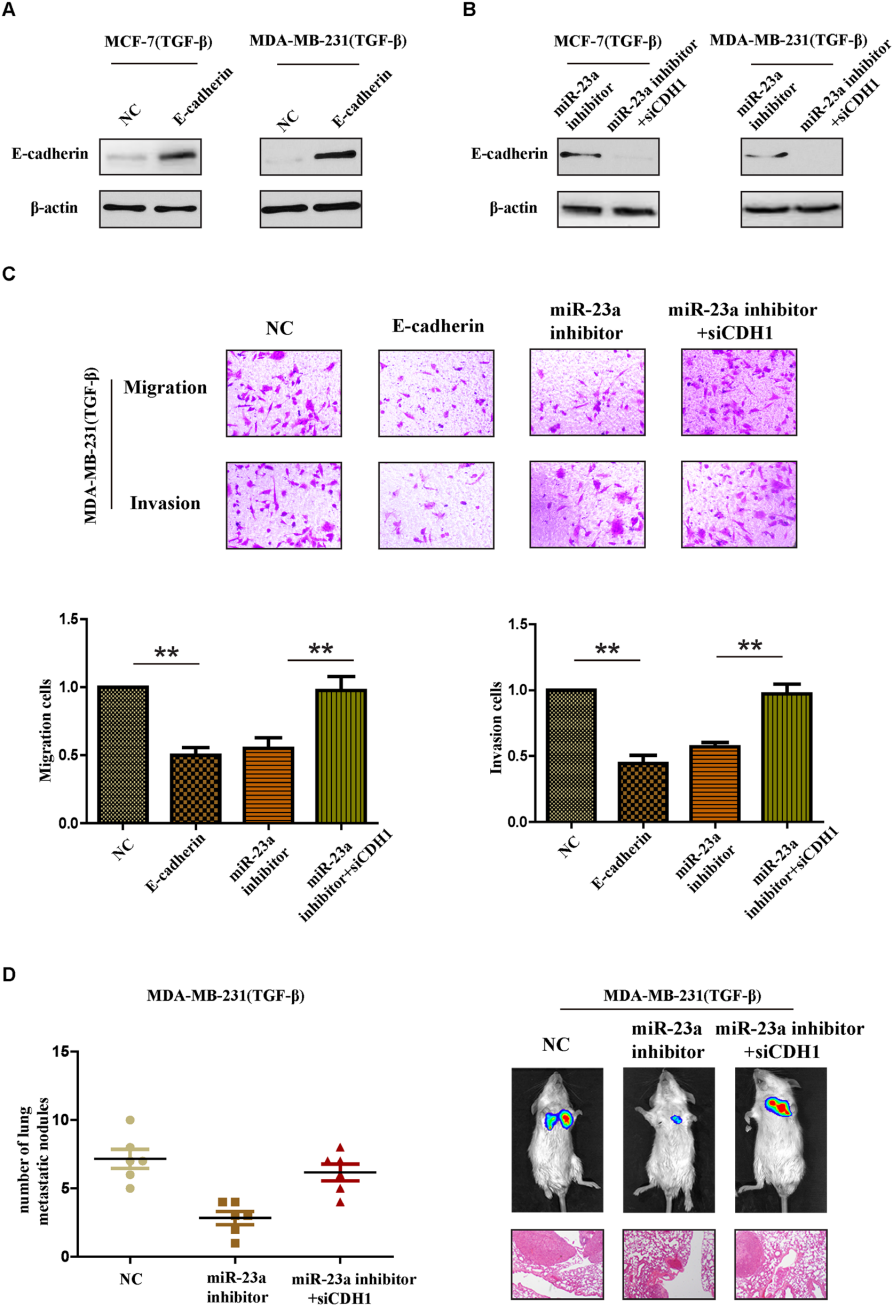
CDH1 critically contributes to the pro-metastatic function of miR-23a in breast cancer cells with TGF-β1 treatment **(A)** Western blot analysis confirmed the transfection of constructs containing CDH1 ORF in cells treated with TGF-β1. **(B)** Western blot analysis confirmed the transfection of specific siRNA in miR-23a-silenced cells treated with TGF-β1. **(C)** Reintroduction of E-cadherin in TGF-β1-treated MDA-MB-231 cells abrogated TGF-β1-induced cell invasion. MiR-23a inhibitor reduced the invasion and migration cells treated by TGF-β1 and siCDH1 restored the cell invasion and migration in miR-23a-silenced cells. **(D)** Tumor metastasis analysis. The number of metastatic lung nodules in each group of nude mice (n= 6 per group, left panel). Representative photos of nude mice injected with indicated cells and micrographs of HE staining of metastatic tumor tissues (right panel). *P<0.05, **P<0.01.

To further confirm whether miR-23a acted its pro-metastatic function through targeting CDH1 in TGF-β1 treated cells, we performed a series of restoration assays using MCF-7 and MDA-MB-231 cells. Construct containing the CDH1 ORF and siRNA were respectively used to increase or decrease E-cadherin expression in breast cancer cells (Figure 5A and 5B). As shown in Figure 5C, miR-23a inhibitor significantly reduced the invasion and migration cells treated by TGF-β1 and siCDH1 could restore the cell invasion and migration in miR-23a-inhibited cells. Meanwhile, the reintroduction of E-cadherin in TGF-β1-treated MDA-MB-231 cells at least partly abrogated TGF-β1-induced cell invasion. Similar results were also confirmed *in vivo* tumor metastasis model. Silencing CDH1 in miR-23a-inhibited cells with TGF-β1 treatment markedly increased the number of lung metastasis nodes (Figure 5D). Taken together, the results strongly demonstrated that miR-23a acted its pro-metastatic function by targeting CDH1.

### MiR-23a targeted CDH1 to hyperactivate Wnt/β-catenin signaling and subsequently mediated the TGF-β1-induced EMT and tumor invasion in breast cancer

**Figure 6:**
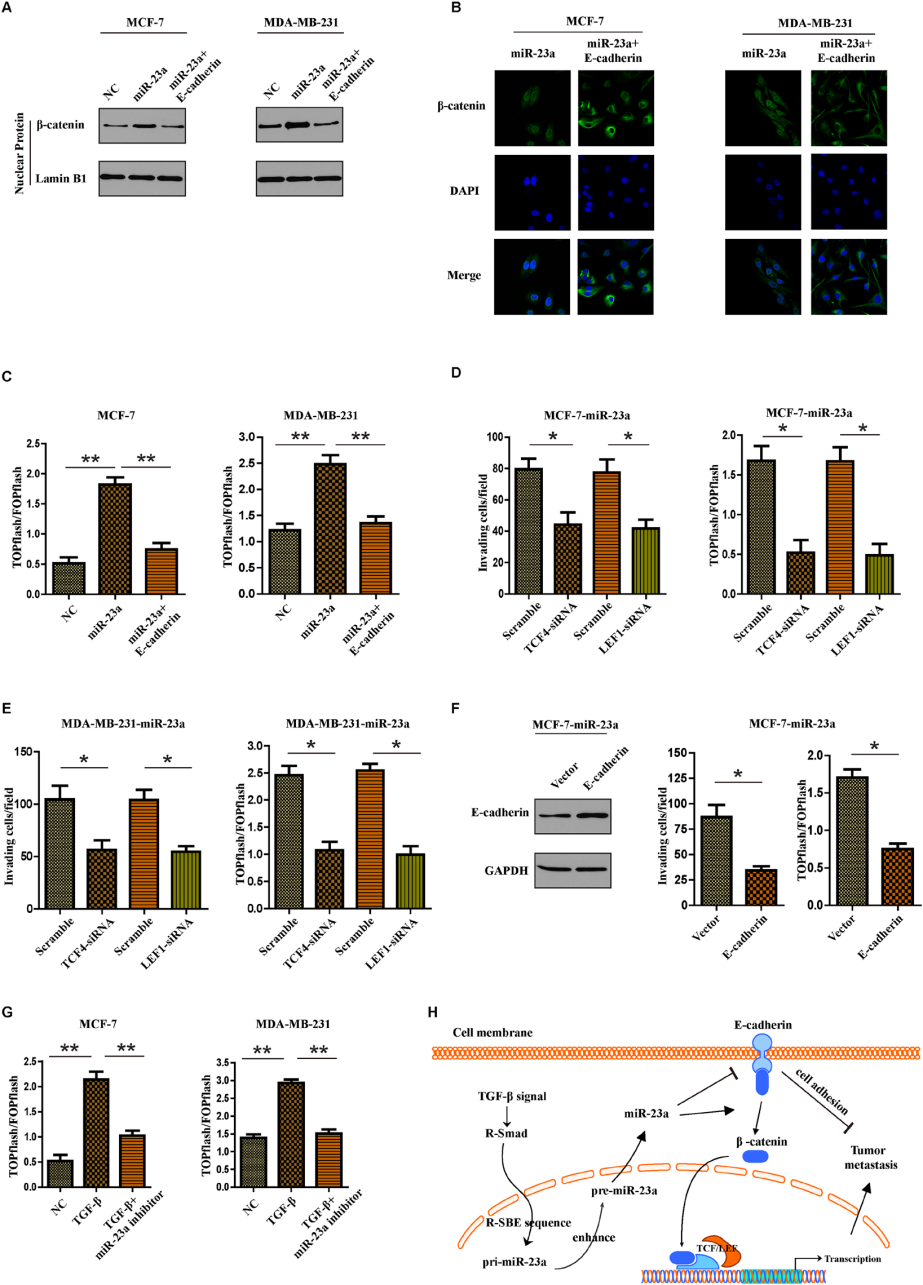
MiR-23a targets CDH1 to hyperactivate Wnt/β-catenin signaling and subsequently mediates the TGF-β1-induced EMT and tumor invasion in breast cancer **(A)** Nuclear fraction of indicated cells was analyzed by Western blot. Lamin B1 was used as a loading control. **(B)** Beta-catenin localization in indicated cells was detected by immunofluorescence staining. **(C)** Indicated cells were transfected with TOPflash and Renilla pRL-TK plasmids, and subjected to dual –luciferase assays 48h after transfection. Reporter activity was normalized to Renilla luciferase activity. **(D** and **E)** Transwell assays were used to detect the quantification of invading cells transfected with indicated siRNAs and luciferase-reported TCF/LEF transcriptional activity in indicated cells were examined. **(F)** Western blot analysis was used to confirm the transfection of constructs containing CDH1 ORF in miR-23a overexpressed MCF-7 cells (left panel). Quantification of invading cells transfected with indicated construct (middle panel) and luciferase-reported TCF/LEF transcriptional activity in indicated cells (right panel). **(G)** Luciferase-reported TCF/LEF transcriptional activity in NC, cells treated with TGF-β1 or miR-23a silenced-cells treated with TGF-β1. **(H)** Schematic representation of a model for the role of miR-23a in the TGF-β-induced tumor metastasis in breast cancer. *P<0.05, **P<0.01.

Given that β-catenin can bind to the cytoplasmic region of E-cadherin and remain in the cytoplasm, we questioned whether miR-23a-induced E-cadherin dysregulation might lead to activation of Wnt/β-catenin signaling and promoted invasion of breast cancer cells. Firstly subcellular fraction assays indicated that upregulation of miR-23a in breast cancer cells resulted in nuclear accumulation of β-catenin, but overexpression of E-cadherin abrogated the effect (Figure 6A). The same results were also confirmed by immunofluorescence staining assays (Figure 6B). Notably, miR-23a overexpression significantly increased the activity of β-catenin, as demonstrated by β-catenin reporter assay. However, overexpression of E-cadherin decreased the activity of Wnt/β-catenin signaling in breast cancer cells (Figure 6C). To further evaluate the role of β-catenin in miR-23a-induced cell invasion, we used siRNA to knock down TCF4 or LEF1 in miR-23a transduced cells. We found that inhibition of β-catenin signaling markedly reduced the invasion ability of miR-23a-transfected cells (Figure 6D and 6E). Moreover, the reintroduction of E-cadherin in miR-23a-transduced MCF7 cells abrogated miR-23a-induced cell invasion and activation of Wnt/β-catenin signaling (Figure 6F). We finally determined if miR-23a mediated the TGF-β-induced activation of Wnt/β-catenin pathway. As shown in Figure 6G, miR-23a inhibitor markedly restored the enhancement of invasion ability and β-catenin pathway activity in cells treatment with TGF-β1. To conclude, miR-23a activated Wnt/β-catenin signaling and subsequently mediated the TGF-β1-induced EMT and tumor invasion in breast cancer by targeting CDH1.

## DISCUSSION

Breast cancer deaths are mostly related to the development of metastasis, which is the characteristic of neoplastic progression. The process of metastasis involves many sequential steps: cells escape from the local primary tumor to enter body circulatory system, evade the immune attacks, survive until they arrive at a secondary organ, undergo extravasation and initiate either intravascular or extravascular proliferation at ectopic sites under the stimulus of local growth factors [20, 21]. Although the precise molecular mechanism of each step is still unclear, epithelial-mesenchymal transition (EMT) is thought to be necessary for the progression of tumor cells to invasion and metastasis [22]. It is universally known that TGF-β is a multifunctional cytokine and potent inducer of EMT in breast cancer [23]. But the mechanism how TGF-β-induced EMT shuts the tumor suppressive function and promotes tumor progression remains a controversy. Hereby we reported that miR-23a regulated TGF-β1-induced EMT and tumor metastasis in breast cancer cells by targeting CDH1 and subsequently activating Wnt/β-catenin signaling.

MicroRNAs were found to regulate gene expression in a sequence-specific fashion and to be dysregulated in multiple cancers, including breast cancer [24]. The crosstalk between miRNAs and TGF-β partly contributes to the mystery of TGF-β switching roles from tumor suppressor to metastasis promoter [25]. For example, miR-99a/b, miR-128-2 and miR-494 can be regulated by TGF-β and subsequently target downstream signal pathways to exert influence on tumor progression. It was also reported that miR-23a could be induced by TGF-β in an SMAD-dependent way and regulate the TGF-β-induced EMT in lung cancer [26]. Consistent with this study, we found TGF-β1 could induce the processing of pre-miR-23a and upregulate miR-23a expression post-transcriptionally in breast cancer. Furthermore, we determined the binding domain for the association between miR-23a and SMAD. The R-SBE sequence of miR-23a was essential for the association with Smad MH1 domain which elucidated a precise regulatory mechanism of TGF-β-induced microRNA.

In the process of TGF-β-induced EMT, E-cadherin is an important regulator and it can be affected by multiple microRNAs through different mechanisms. It was reported that miR-9 might initiate TGF-β-induced EMT and promote tumor metastasis in breast cancer by targeting the mRNA of E-cadherin [27]. On the other hand, E-cadherin was also known to be transcriptionally regulated by some factors, such as ZEB1, ZEB2, Twist1 and SNAIL. MiR-200 family members were reported to directly target the mRNA of ZEB1 and ZEB2, subsequently increase the E-cadherin transcription [28]. Herein we reveal a new mechanism that miR-23a contributes to the process of TGF-β-induced EMT by targeting the mRNA of E-cadherin. MiR-23a acts as a media between TGF-β and E-cadherin. Meanwhile, the sequences of miR-23a that bind to TGF-β and E-cadherin were found respectively which made the results more convincing.

Wnt/β-catenin signaling pathway has been found aberrantly activated and contributing to the progression of breast cancer [29]. The β-catenin protein rarely mutates in breast cancer, but the localization of β-catenin in breast cancer cells often abnormally changes. It is known that E-cadherin can bind to β-catenin soon after it is synthesized in the cytoplasm and the two proteins translocate together to the membrane, recruit the actin-binding protein, thereby linking adherens junctions to the actin cytoskeleton [15]. Given the background, we detected if the dysregulation of E-cadherin induced by miR-23a could lead to the freedom of β-catenin and subsequently promote the nuclear translocation of β-catenin. In line with our speculation, we found that miR-23a indeed activated Wnt/β-catenin signaling by targeting CDH1 and promoted the TGF-β1-induced EMT and tumor invasion in breast cancer. We reveal a new regulatory mechanism that miR-23a mediates the TGF-β1-induced EMT and tumor invasion in breast cancer by targeting CDH1 and activating Wnt/β-catenin signaling.

## MATERIALS AND METHODS

### Cell lines and tissue specimens

Human breast cancer cell lines, MCF-7, MDA-MB-468, T47D, BT-549 and MDA-MB-231 were incubated under the conditions following ATCC instructions. All of the media purchased from Hyclone were supplemented with 10% FBS (Gibco). From January 2015 to December 2016, 30 pairs of breast cancer samples were obtained Harbin Medical University Cancer Hospital. The specimens were used for RNA extraction or *in situ* hybridization. All human samples were gathered with informed consent and the study was approved by the ethics committee of Harbin Medical University.

### RNA isolation and RT-PCR

Total RNA extraction and reverse transcription were performed as previously described (10). RT-PCR was performed by using Platinum Taq DNA Polymerase (Invitrogen). The PCR primers for miR-23a and U6 were purchased from GenePharma (China). Human specific primers for miR-23a: Forward primer: 5’- CAGGCGGGTAGTAGATG-3’, miR-23a reverse primer: 5’-AGGGACGGGCATGGAAAGG-3’. U6 forward primer: 5’-CTCGCTTCGGCAGCACA-3’, U6 reverse primer: 5’-AACGCTTCACGAATTTGCGT-3’. Pri-miR-23a forward primer: 5’-CCTCACCCCTGTGCCACG-3’, reverse primer: 5’-AGCATCCTCGGTGGCAGA-3’. Pre-miR-23a forward primer: 5’-TTCCTGGGGATGGGATTT-3’, reverse primer: 5’-TCAGGGTCGGTTGGAAATC-3’. The level of CDH1 mRNA was detected using the forward primer, 5’-AATGCCGCCATCGCTTAC-3′ and reverse primer, 5′-TCAGGCACCTGACCCTTGTA-3′. All the primers for pri- and pre-miR-23a (wild type or mutants) were purchased from GenePharma. PCR cycling conditions were: Stage I: 94°C for 3 min, 54°C for 50 sec, 72°C for 30 sec (2 cycles); Stage II: 94°C for 3 min, 54°C for 50 sec, 72°C for 30 sec (50 cycles); Stage III: 72°C for 5 min.

### GST-SMAD-RNA pull down assays

GST-Smad fusion protein was expressed in E.coli, isolated, and subsequently bound to glutathione S-sepharose beads. After purification, Smad bound beads were washed 3X5min at 4°C in wash buffer (10mM Tri-HCI pH 7.6, 0.1% triton, .5M LiCl) and 1X 10’ at 4 °C in binding buffer (20mM Tris-HCl pH 7.6, 0.1% Tween-20, .1M KCl, 0.1% Triton). After the addition of 10 pmoles synthesized mature miRNA or ∼20 pmoles *in vitro* transcribed 112bp pri-miR-23a, beads were resuspended in 100μl binding buffer and incubated with 1ng poly-[dI-dC], 25μg tRNA and 2μl RNAse inhibitor for 10 minutes. Following 1 hr incubation at 4°C, beads were washed 3X with buffer. Elutions of bound RNA were washed by Elution buffer (0.15M NaCl, 1% SDS) at room temperature for 20 min. 600 ml Trizol-LS (Invitrogen) was mixed and the RNA was purified. The purified RNA was then subjected to quantitative real time PCR to detect relative levels of RNA binding to Smad-GST fusion proteins.

### Immunofluorescence assays

Breast cancer cells were fixed with 4% formaldehyde and then permeabilized 0.2% Triton X-100 for 15 min at room temperature. After blocking, cells were incubated with indicated primary antibodies and corresponding secondary antibodies. Confocal images were taken using Zeiss confocal microscope.

### *In situ* hybridization

To detect the expression of miR-23a in breast cancer tissues, we performed *in situ* hybridization by using Biochain Kit (Biochain IsHyb *In Situ* hybridization kit) following the manufacturer’s protocol. Tissues were deparaffinized and fixed, digested with 0.1% Triton-X. Tissues were then incubated with the AP-conjugated anti-digoxingenin antibody. Further, reacted with NBT/BCIP (Pierce) followed by nuclear fast counterstaining, the slides were analyzed under microscope.

### Transfection and treatment with TGF-β1

MiR-23a mimics, scrambled miR-control, CDH1 siRNA or siRNA negative control, purchased from GenePharma (China), were transfected into 70% of the indicated cells according to the manufacturer’s protocol. CDH1 Human cDNA ORF Clone was purchased from Origene (Origene Technologies). The pri-miR-23a sequence was constructed by annealing the following oligonucleotides: 5’-tcgagccccctcacccctgtgccacGGCCGGCTGGGGTTCCTGGGGATGGGATTTGCTTCCTGTCACAAATCACATTGCCAGGGATTTCCAACCGACCctgagctctgccaccgaggac-3’,5’-tcgagtcctcggtggcagagctcagGGTCGGTTGGAAATCCCTGGCAATGTGATTTGTGACAGGAAGCAAATCCCATCCCCAGGAACCCCAGCCGGCCgtggcacaggggtgagggggc-3’. The M1-M3 sequence were designed and chemically synthesized similarly. Transfections were performed using Lipofectamine 2000 (Invitrogen) according to the manufacturer’s protocol. After 24 hrs of transfection, cells were treated with TGF-β1 (Cell Signaling Technology) at a concentration of 10ng/ml in serum-free medium for indicated time periods.

### Migration and invasion assay

6.5-mm diameter Boyden chambers with a pore size of 8.0 μm (Corning) was used for migration and invasion assays. Briefly, the cells were resuspended in the FBS-free medium and placed in the upper transwell chambers coated with fibronectin on the lower surface. The lower chamber was filled with 500 μl medium containing 30% FBS as a chemoattractant. Cells were fixed in 4% formaldehyde and stained with 0.1% crystal violet after 24 hrs. Five Random fields were calculated under a light microscope. However, cell invasion (2.0 × 105 cells per well) was evaluated in 24-well Matrigel-coated invasion chambers after 48 hours.

### Wound healing assays

Breast cancer cells were plated into 6-well plate. Once the cells nearly achieved 100% confluence, a 200 ul pipette tip was used to create a scraped line. Then the cells were cultured in new medium for 24 hrs. The speed of wound closure was imaged with a microscope and the rate of closure was assessed.

### Dual-luciferase reporter assay

Dual-luciferase 3' UTR reporter assay was conducted to validate CDH1 as a direct target of miR-23a. Wild-type or mutant of 3' untranslated region (UTR) sequences of CDH1 was inserted into the Fse I and Xba I sites of the pGL3 vector. HEK293T cells infected with miR-23a lentivirus or NC lentivirus were seeded into 96-well plates. Luciferase reporter plasmid was cotransfected with 10 ng of pRL-TK vector into cells by Lipofectamine LTX (Invitrogen). After twenty-four hours, cells were harvested and firefly and Renilla luciferase activity was detected using Dual-luciferase Reporter Assay System Kits (Promega). Additionally, the mutant CDH1 3’UTR reporter was made by site-directed mutagenesis in the putative target site of miR-23a using Quickchange XL site-directed mutagenesis kit (Agilent Technologies). The reporter plasmids containing TOPflash or mutated FOPflash TCF/LEF DNA binding sites were purchased from Upstate Biotechnology.

### *In vivo* tumorigenic assay

Animal experiment protocol was approved in accordance with NIH guidelines for the use of experimental animals. Male NOD/SCID mice, 6 per group, obtained from the Beijing Vital River Laboratory Animal Technology, were injected with the indicated cells (2.0 × 106) through the tail vein. After four weeks, mice were injected with luciferin 10 minutes before imaging. Bioluminescence imaging was performed by the Xenogen IVIS Spectrum Imaging System (Caliper Life Sciences, Hopkinton, MA, USA). Then the mice were sacrificed and lungs were isolated for detection of the number of metastatic nodules. Tumor volume (V) was estimated. For each tissue, haematoxylin and eosin (HE) staining was performed. All animal experimental procedures were approved by Harbin Medical University Experimental Animal Care Commission.

### Western blot

Indicated cells were washed with PBS and then lysed. Cell protein lysates were electrophoresed through 8% SDS PAGE and then transferred to a polyvinylidene difluoride membrane. The membranes were blocked incubated with the primary antibodies below: E-cadherin (Cell Signaling Technology), β-actin, vimentin (Cell Signaling Technology), GAPDH and β-catenin (Cell Signaling Technology). Nuclear proteins were extracted using the Nuclear Extraction Kit (Active Motif) according to the manufacturer’s instructions.

### Statistical analysis

Results were expressed mean ± standard deviation (S.D.) from at least three independent experiments. The Student’s t-test was applied to compare the differences between groups. Statistical analysis was performed by SPSS 17.0 software. Significant results are indicated by asterisks P<0.05 (*), P<0.01(**).

## Abbreviations

MiR-23a: microRNA-23a, epithelial-mesenchymal transition
EMT: RNA Smad binding element
R-SBE: 3’-untranslated region
UTR: receptor-specific SMAD
